# 2165: Effects of a novel 2-phase rehabilitation program on postural control in older adults: A pilot study

**DOI:** 10.1017/cts.2017.117

**Published:** 2018-05-10

**Authors:** Evan Papa, Mahdi Hassan, Sandra Hunter, Rita Patterson, Nicoleta Bugnariu

## Abstract

OBJECTIVES/SPECIFIC AIMS: Falls are a major source of morbidity and disability in the aging population. Twenty to thirty percent of older adults who fall suffer moderate to severe injuries such as lacerations, hip fractures, and head traumas. A serious component of falling often overlooked is the fear of falling. The fear of falling is part of a debilitating spiral that leads to decreased activity and muscle weakness. The goal of this investigation was to determine if a novel 2-phase rehabilitation program designed to reduce the fear of falling and increase muscle strength could improve postural control during falls in older adults with balance impairments. METHODS/STUDY POPULATION: Four older adults participated in 8 cognitive restructuring workshops entitled A Matter of Balance (AMOB): 2 hours/week, total of 16 hours, designed to restructure thought patterns relative to falls and reduce the fear of falling. Within 1–2 weeks of completion, participants enrolled in Phase II: a standardized 10-week lower-extremity strengthening program. Participants performed high-intensity concentric resistance exercise on a modified seated ergometer (Eccentron, BTE Technologies) twice per week for up to 20 minutes per session. Fear of falling was assessed using the Activities-Specific Balance Confidence (ABC) scale. Postural control was assessed during reproducible falls at 3 phases: baseline (T0), after Phase I AMOB (T1), and after Phase II strengthening (T2). Falls were induced by treadmill perturbations (VGait system, MotekForce Link) occurring at slow and fast belt accelerations. A 3×3 ANOVA was conducted on postural control outcomes with phase and stepping cycle as independent factors. Pairwise comparisons were analyzed with the Bonferroni correction. RESULTS/ANTICIPATED RESULTS: Statistically significant main effects were found for phase and stepping cycle (*p*=0.003, *p*=0.00). No statistically significant interaction effects were found. However, a trend toward increasing Center of Pressure-Center of Mass (COP-COM) distance occurred after each intervention phase (T1 and T2) during fast treadmill perturbations. The greatest increase in COP-COM distance was found at 100% of the stepping cycle during fast perturbations following 10 weeks of resistance training compared with baseline (*p*=0.006). No significant differences were found in fear of falling between phases (*p*=0.682). DISCUSSION/SIGNIFICANCE OF IMPACT: A large COP-COM distance suggests the individual is able to allow straying of the COM outside of the functional base while recovering balance. Meanwhile, a small COP-COM distance represents a conservative approach to postural tasks, in that the performer does not feel stable enough to allow separation of the COP and COM. These pilot data suggest that a 2-phase rehabilitation program can improve specific components of postural control during recovery from falls. Rehabilitation interventions aimed at reducing falls in older adults should consider adding a component of cognitive restructuring in conjunction with standard of care resistance training.

